# Functionally distinct patterns of nucleosome remodeling at enhancers in glucocorticoid-treated acute lymphoblastic leukemia

**DOI:** 10.1186/s13072-015-0046-0

**Published:** 2015-12-02

**Authors:** Jennifer N. Wu, Luca Pinello, Elinor Yissachar, Jonathan W. Wischhusen, Guo-Cheng Yuan, Charles W. M. Roberts

**Affiliations:** Department of Pediatric Oncology, Dana-Farber Cancer Institute, Boston, MA USA; Department of Biostatistics and Computational Biology, Dana-Farber Cancer Institute and Harvard T. H. Chan School of Public Heath, Boston, MA USA; SBH Sciences Inc., Natick, MA USA; Morsani College of Medicine, Tampa, FL USA; Department of Oncology, St. Jude Children’s Research Hospital, 262 Danny Thomas Place, MS 281, Memphis, TN 38105 USA

**Keywords:** Chromatin remodeling, Nucleosome, Glucocorticoid receptor, Transcription enhancer, Transcription regulation, ChIP-seq

## Abstract

**Background:**

Precise nucleosome positioning is an increasingly recognized feature of promoters and enhancers, reflecting complex contributions of DNA sequence, nucleosome positioning, histone modification and transcription factor binding to enhancer activity and regulation of gene expression. Changes in nucleosome position and occupancy, histone variants and modifications, and chromatin remodeling are also critical elements of dynamic transcriptional regulation, but poorly understood at enhancers. We investigated glucocorticoid receptor-associated (GR) nucleosome dynamics at enhancers in acute lymphoblastic leukemia.

**Results:**

For the first time, we demonstrate functionally distinct modes of nucleosome remodeling upon chromatin binding by GR, which we term central, non-central, phased, and minimal. Central and non-central remodeling reflect nucleosome eviction by GR and cofactors, respectively. Phased remodeling involves nucleosome repositioning and is associated with rapidly activated enhancers and induction of gene expression. Minimal remodeling sites initially have low levels of enhancer-associated histone modification, but the majority of these regions gain H3K4me2 or H3K27Ac to become de novo enhancers. Minimal remodeling regions are associated with gene ontologies specific to decreased B cell number and mTOR inhibition and may make unique contributions to glucocorticoid-induced leukemia cell death.

**Conclusions:**

Our findings form a novel framework for understanding the dynamic interplay between transcription factor binding, nucleosome remodeling, enhancer function, and gene expression in the leukemia response to glucocorticoids.

**Electronic supplementary material:**

The online version of this article (doi:10.1186/s13072-015-0046-0) contains supplementary material, which is available to authorized users.

## Background

Glucocorticoids have been mainstays of treatment for a variety of malignant, autoimmune, and inflammatory diseases for many decades. Glucocorticoids are critical components of therapy for acute lymphoblastic leukemia (ALL), and resistance to glucocorticoid-induced cell death is associated with poor prognosis in childhood ALL [1, 2]. Dexamethasone is a widely used synthetic glucocorticoid that binds to the glucocorticoid receptor (GR) in the cytosol and induces receptor dimerization and nuclear translocation. Like other nuclear hormone receptors such as androgen receptor (AR) and estrogen receptor (ER), ligand-bound GR engages chromatin and acts as a transcription factor. The signaling and transcriptional responses of lymphoid cells to glucocorticoids have been subject to intensive study, yet the chromatin changes linking GR binding to transcriptional changes remain incompletely understood. We set out to explore the dynamics of dexamethasone-induced chromatin remodeling at enhancers in the RS4; 11 B cell ALL cell line.

Enhancers are generally defined as non-promoter DNA elements that contribute to modulation of gene expression. The human genome is estimated to harbor hundreds of thousands enhancers, and the enhancers participating in transcriptional regulation differ extensively among cell types and stages of differentiation. Among the many unresolved questions surrounding enhancers are those of how nucleosome positioning and chromatin remodeling contribute to their modulation of gene expression [3]. In mammalian cells, nucleosome positioning has largely been studied at promoters. The transcriptional start sites of actively transcribed genes are characterized by a nucleosome free region. This nucleosome-free region is flanked by short arrays of 3–5 well-positioned nucleosomes, often described as phased [4, 5]. More recently, Gaffney et al. have described well-positioned nucleosome arrays flanking transcription factor binding sites within mammalian enhancers [6, 7]. In comparison, nucleosome dynamics remain far more mysterious. Although often depicted as tiny spools around which DNA is wound, nucleosomes bind DNA transiently [8]. The likelihood that a given segment of DNA is nucleosome-occupied at any given moment is thought to be affected by many factors, including DNA sequence, transcription factor binding, histone modifications and variants, as well as ATP-dependent chromatin remodeling [9, 10]. One obstacle to better understanding nucleosome dynamics is the size of mammalian genomes. High-resolution, genome-wide nucleosome positioning studies typically utilize micrococcal nuclease (MNase) digestion of total native chromatin, followed by deep sequencing of resulting DNA fragments [11]. The number of sequencing reads required by this method remains prohibitively high for experiments aimed at comparisons of nucleosome position and occupancy among multiple conditions or at individual genomic locations.

Many groups have circumvented this difficulty by measuring chromatin “accessibility” typified by hypersensitivity to cleavage by DNaseI (DHS) or insertion of a transposon (ATAC-Seq). These techniques require far fewer sequencing reads than MNase-Seq, and chromatin accessibility is often used to approximate decreased nucleosome occupancy with relatively low resolution and sensitivity. Although DHS offers limited insights into nucleosome dynamics, it identifies active enhancers and promoters very well. Increased DHS is a widely accepted indicator of enhancer activation, and many groups have developed computational models that use changes in DHS to predict transcription factor binding [12–15].

Active enhancers are also characterized by histone modifications that include histone 3 lysine 4 mono- and di-methylation (H3K4me1 and H3K4me2), and H3K27 acetylation [16, 17]. He et al. employed chromatin immunoprecipitation of H3K4me2 and sequencing (ChIP-Seq) to interrogate nucleosome dynamics associated with AR and ER binding to chromatin [12, 18]. Like DHS, ChIP-Seq requires relatively small numbers of sequencing reads to provide genome-wide information about active enhancers. Moreover, ChIP-Seq performed on MNase-digested native chromatin affords a high-resolution assessment of nucleosome position that is well suited to studying nucleosome dynamics. Nucleosome remodeling has long been known to be a critical component of GR-mediated transcriptional modulation. GR binds directly to the SWI/SNF chromatin remodeling complex, and the ATP-dependent activity of SWI/SNF is necessary for maximal transcriptional activation by GR [19, 20]. Inhibition of the SWI/SNF ATPase (SMARCA4) reveals both SMARCA4-dependent and -independent glucocorticoid-induced DHS and gene expression changes, suggesting diverse remodeling requirements [21, 22].

We wished to better understand nucleosome dynamics and their relationships to transcription factor binding, enhancer activity, and gene expression. We performed ChIP-Seq for GR and multiple enhancer-defining histone modifications, as well as DHS and gene expression analysis both before and after the addition of dexamethasone. Using a number of open-source computational packages, we have developed an analytic approach to ChIP-Seq that captures rapid changes in nucleosome position and occupancy associated with chromatin binding by GR. We find that nucleosome remodeling is highly variable among GR binding sites, but can be computationally resolved into four predominant modes that we have dubbed “central”, “non-central”, “phased”, and “minimal”. Moreover, each class of nucleosome remodeling represents functionally distinct contributions to enhancer activation and changes in gene expression. We propose that patterns of nucleosome remodeling offer important new insights into dynamic changes of the enhancer landscape and transcriptional outcomes following glucocorticoid treatment in ALL.

## Results and discussion

### GR binding is not restricted to enhancers or DHS sites

**Fig. 1:**
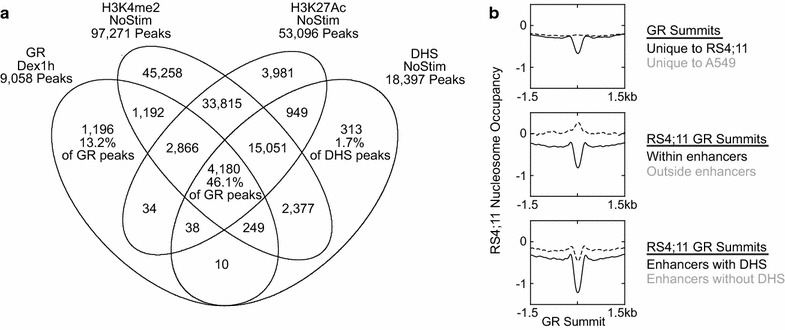
GR binding relative to enhancers and nucleosome-depleted regions. **a** Venn diagram illustrating the binding of GR peaks within the enhancer landscape of unstimulated cells (labeled as NoStim). Of the 9058 GR peaks detected by ChIP-Seq after 1 h of dexamethasone stimulation, 45 % bind regions without prior DHS, and 13 % bind regions lacking any prior enhancer marks. **b** Signal profiles of nucleosome occupancy (normalized signal of MNase-digested ChIP-Seq input DNA) in a 3-kb window surrounding GR summits

We first sought to identify enhancers in the RS4; 11 acute lymphoblastic leukemia cell line, and to characterize GR binding with respect to this existing chromatin landscape. We performed ChIP-Seq of GR and three different chromatin features typically used to define enhancers (H3K4me2, H3K27Ac, and DHS). Cells were cultured in charcoal-dextran-treated serum for 2 days prior to each experiment, effectively depriving them of hormone exposure. GR ChIP-Seq was performed on fixed and sonicated chromatin, whereas histone modification ChIP-Seq was performed on native chromatin fragmented by MNase digestion. As expected, very few GR–chromatin interactions were detected in cells not treated with dexamethasone. In contrast, 1 h after treatment with 10 nM dexamethasone, 9058 GR binding events were detected, with virtually no overlap between binding sites in the unstimulated and dexamethasone-stimulated cells. As in other cell types, promoter-bound GR peaks in RS4; 11 are a small minority (5.3 %). Among GR binding sites, we found that 46 % occur at enhancers having all three marks. We find that 45 % of GR binding events in RS4; 11 cells occur at sites not having pre-existing DHS. Additionally, 13 % of GR binding occurs at sites bearing none of the canonical enhancer marks, where GR may function as a true pioneer factor (Fig. 1a). Prior studies have suggested that accessible chromatin (DHS) is largely required for GR to bind [21–23]. Many different experimental conditions and computational methods have been used to measure DHS, and disparities between our findings and those of other groups may simply reflect this methodologic variability.

### Decreased nucleosome occupancy precedes GR binding within enhancers

We wished to examine how nucleosome occupancy, in addition to DNA motif and enhancer chromatin marks, contributes to determining where GR will bind. Using the MNase-digested input DNA from our histone modification ChIP-Seq experiments, we find that average nucleosome occupancy in hormone-deprived cells is specifically decreased at sites that will subsequently bind GR upon dexamethasone exposure. To examine the cell type specificity of this localized nucleosome depletion, we used publicly available GR ChIP-Seq data for the A549 lung carcinoma cell line from the ENCODE project [24–26] (filename at UCSC Genome Browser: wgEncodeAwgTfbsHaibA549GrPcr1xDex50nmUniPk.narrowPeak.gz). On average, GR binding sites unique to A549 do not show nucleosome depletion in RS4; 11 (Fig. 1b, top panel). Notably, decreased nucleosome occupancy characterizes GR binding sites within enhancers (having H3K4me2 and/or H3K27Ac), but not those that will occur at sites that lack pre-existing enhancer marks, which are actually characterized by increased nucleosome occupancy centered over the future GR binding site (Fig. 1b, middle panel). We next asked whether the nucleosome depletion seen at enhancer GR binding sites is restricted to the DHS sites. Globally, enhancers demonstrate below-average nucleosome occupancy, consistent with an “open” state that is more sensitive to MNase digestion. We found that, on average, sites without DHS have a small degree of focal nucleosome depletion prior to GR binding (Fig. 1b, bottom panel). Nucleosome occupancy is lower at sites of DHS, not just centrally, but for >1.5 kb flanking the GR binding site, consistent with broader nucleosome exclusion/eviction.

### Modified ChIP-Seq analysis

**Fig. 2:**
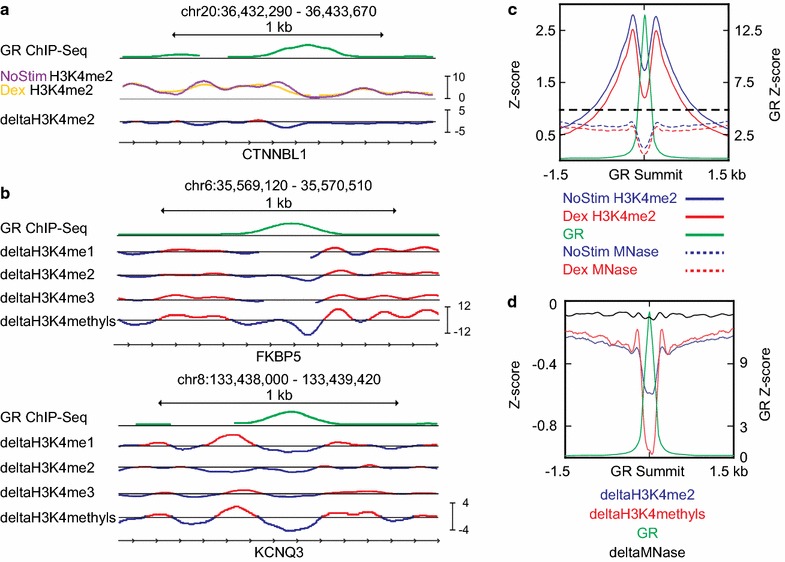
Definition and validation of deltaH3K4methyls signal. **a** Snapshot demonstrating how deltaH3K4me2 track is defined—normalized H3K4me2 ChIP-Seq signal from unstimulated cells is subtracted from that of dexamethasone-stimulated cells, emphasizing the pattern of ChIP-Seq signal change at this GR binding site. **b** Snapshots demonstrating how the deltaH3K4methyls track is defined. At many sites, the summed signal has the same shape as its components (*upper panel*), while at others the deltaH3K4methyls track resolves discordancies between component signals (*lower panel*). **c** Aggregate signal profile of normalized H3K4me2 ChIP-Seq signal from unstimulated (*solid blue*) and dexamethasone-stimulated (*solid red*) cells in a 3-kb window surrounding all GR summits. Normalized MNase-Seq signal from unstimulated (*dashed blue*) and dexamethasone-stimulated (*dashed red*) cells is overlaid. **d** Aggregate signal profile of deltaH3K4me2 (*blue*) versus deltaH3K4methyls (*red*) versus deltaMNase (*black*) in a 3-kb window surrounding all GR summits

We next sought to examine the dynamics of nucleosome positioning and occupancy around GR binding sites in RS4; 11 cells. We predicated these analyses on histone modification ChIP-Seq of MNase-digested native chromatin to identify and enrich for enhancer DNA as well as limit the necessary depth of sequencing required to assess nucleosome position. To focus on nucleosome dynamics, we first calculated a “delta” ChIP-Seq signal by subtracting the normalized ChIP signal for unstimulated cells from the normalized ChIP signal for dexamethasone-treated cells. This transformation emphasizes the changes in ChIP signal occurring in response to the hour-long dexamethasone stimulus (Fig. 2a). When we examined the average H3K4me2 signal across a 3-kb window centered on the summit of all GR binding peaks, we found that global ChIP-Seq profiles before and after GR binding closely resemble those reported for AR (Fig. 2c) [18]. The average deltaH3K4me2 signal provides an alternative visualization (Fig. 2d, blue line) demonstrating a central depletion in response to dexamethasone having a width of ~450 bp. This is consistent with previous descriptions of H3K4me2 changes in response to transcription factor binding, which have been described as a nucleosome-depleted region (NDR), and suggests that both our data and our delta signal transformation faithfully recapitulate prior results [12, 18].

Our primary aim was to understand nucleosome remodeling, a term we use to describe changes in nucleosome occupancy, nucleosome positioning, or both. However, a decrease in H3K4me2 signal may result from methylation (i.e., transformation to H3K4me3) or demethylation (i.e., transformation to H3K4me1 or unmethylated H3K4), as well as nucleosome remodeling. In an effort to mitigate the changes in ChIP-Seq signal resulting from histone modification, we calculated the sum of the deltaH3K4me1, deltaH3K4me2, and deltaH3K4me3 signals, henceforth referred to as the deltaH3K4methyls signal. The result of this summation is exemplified in Fig. 2b. At some sites, the deltaH3K4methyls signal has the same general shape as its component signals (Fig. 2b, top panel). Where the deltaH3K4me1, deltaH3K4me2, and/or deltaH3K4me3 tracks show subtle or discordant changes (Fig. 2b, bottom panel), the deltaK4methyls transformation smoothes signal and improves signal-to-noise ratio. The average deltaH3K4methyls signal across GR binding summits has the same shape and width of NDR as the deltaH3K4me2 signal (Fig. 2d, red line).

### Heterogeneous and asymmetric nucleosome remodeling accompanies GR binding

**Fig. 3:**
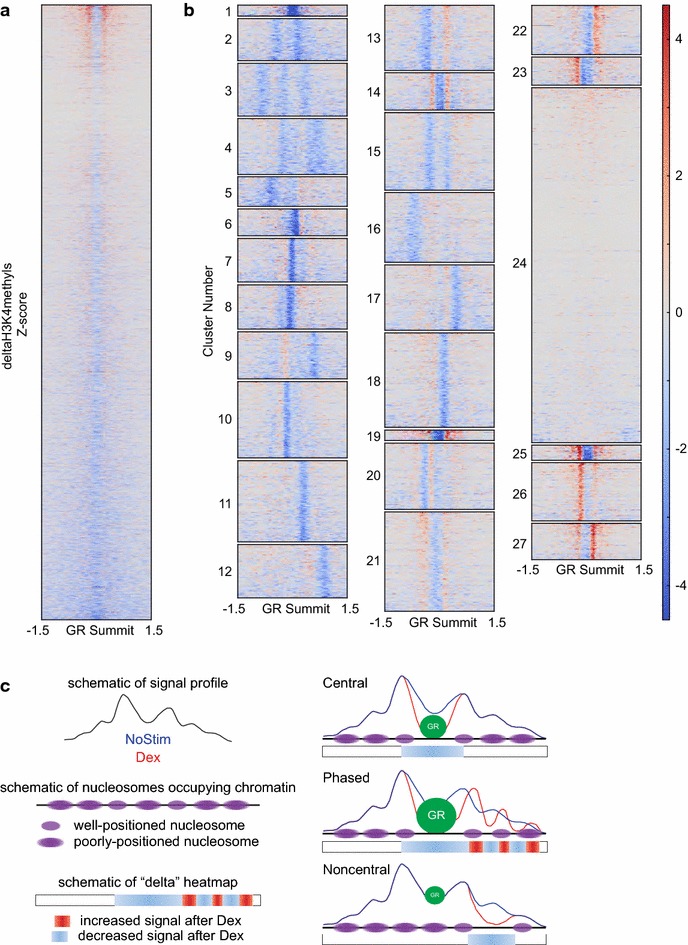
K-means clustering resolves deltaH3K4methyls signal into diverse patterns of nucleosome remodeling. **a** Heatmap of deltaH3K4methyls signal in a 3-kb window surrounding all GR summits. **b** Heatmaps representing the 27 k-means clusters. Color scale for *Z*-score of deltaH3K4methyls signal is shown on the *right*. **c** Schematic representation of proposed patterns of nucleosome remodeling

We next wished to assess the relationship between the deltaH3K4methyls ChIP-Seq signal and changes in nucleosome occupancy. We performed deep sequencing of the MNase-digested input chromatin that was used for our ChIP-Seq (4.5–5 × 10^8^ reads/condition). As seen in Fig. 2c, the normalized MNase signal averaged across all GR binding sites shows decreased central nucleosome occupancy corresponding to the decreased H3K4me2 ChIP-Seq signal in both unstimulated and dexamethasone-stimulated cells. However, the deltaMNase signal (Fig. 2d, black line) has low signal-to-noise ratio. We next examined a heatmap of the deltaH3K4methyls signal (Fig. 3a). This shows the expected loss of ChIP-Seq signal overlying the summit of GR binding peaks. Also apparent is significant variability in the width and position of decreased signal relative to the GR summit. We, therefore, performed *k*-means clustering to partition GR binding sites according to the magnitude and position of change in ChIP-Seq signal. Results of *k*-means clustering (*k* = 27) of the deltaH3K4methyls signal in a 3-kb window centered on the GR binding summit are illustrated in Fig. 3b.

**Fig. 4:**
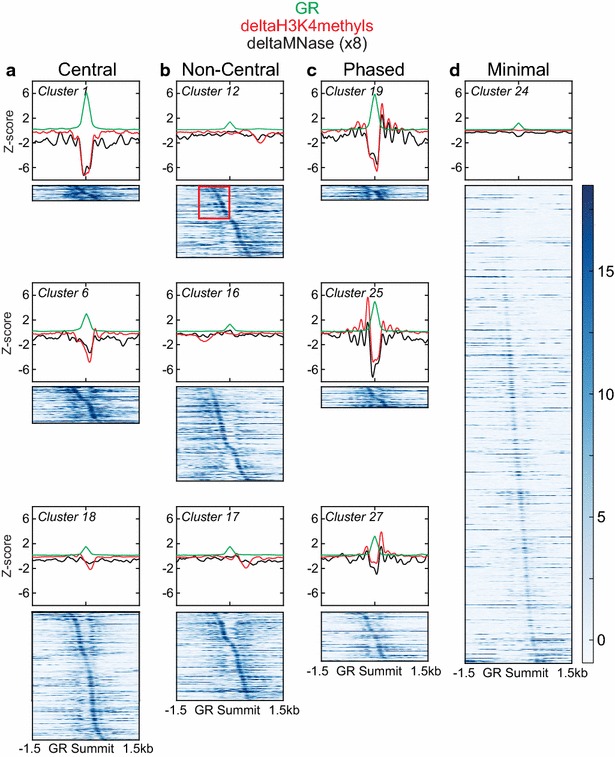
Four distinct modes of nucleosome remodeling. Representative signal profiles of *k*-means clusters belonging to each mode of remodeling (**a** central; **b** non-central; **c** phased; **d** minimal). deltaH3K4methyls (*red*), GR (*green*), and deltaMNase (*black*) signals are overlaid for comparison. deltaMNase signal is magnified eightfold to facilitate visual comparison. Heatmaps illustrate nucleosome positioning prior to GR binding (NoStim H3K4methyls signal). *Red box* overlying heatmap of cluster 12 highlights evidence of well-positioned, phased nucleosomes in the unstimulated condition

As expected, clustering reveals several groups (clusters 1, 6, 7, 8, etc.) distinguished by subtle shifts in the location of their central signal loss. Strikingly, clustering also reveals previously unrecognized patterns of nucleosome remodeling occurring at and near GR binding sites. When these clusters are applied to the deltaMNase signal (Fig. 4), we see that decreased nucleosome occupancy closely mirrors the “nucleosome depleted region” of the deltaH3K4methyls signal.

### Classifying patterns of GR-associated nucleosome remodeling

As seen in Fig. 4, many clusters have a very similar signal shape and are distinguished by position relative to the GR binding summit. We, therefore, re-classified individual clusters into distinct patterns of nucleosome dynamics (illustrated schematically in Fig. 3c), defined as follows. Central remodeling overlaps the GR binding peak and alters the depth and/or width of the NDR (Fig. 4a, signal profiles). Non-central remodeling does not overlap the GR binding peak and affects the occupancy/position of one or more nucleosomes flanking the NDR (Fig. 4b, signal profiles). Phased remodeling occurs at a minority of sites, where distinct and alternating peaks and valleys of nucleosome remodeling flanking the GR peak are apparent (Fig. 4c, signal profiles). Based upon these patterns, we grouped the GR binding sites into four “modes” of remodeling. In the “central mode”, changes in H3K4methyls signal are restricted to the region occupied by GR. Within the “non-central mode”, changes in H3K4methyls are seen in regions not occupied by GR. The “phased mode” shows remodeling of multiple flanking nucleosomes in addition to that overlapping the GR binding peak. The “minimal mode” represents all sites of GR binding not classified as central, non-central, or phased and shows relatively small changes in H3K4methyls (4D, signal profile). Having thus defined these four modes of nucleosome remodeling, we set out to test whether they demonstrate functionally distinct relationships between GR binding, nucleosome remodeling, enhancer activation, and modulation of gene expression.

### Four modes of nucleosome remodeling capture distinct subsets of GR binding and active enhancers

**Fig. 5:**
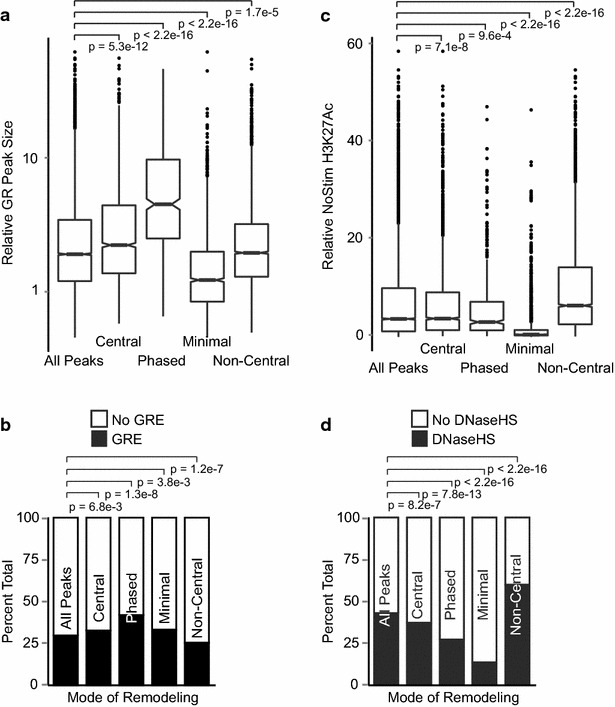
Four modes of remodeling describe distinct subsets of GR peaks and enhancers. **a** *Box and whisker plot* of relative GR peak size reveals significant differences in GR peak size associated with each mode of remodeling. For all *box and whisker plots*, *hinges of box* are at located at 1st and 3rd quartiles. *Whiskers* extend to 1.5x interquartile range (IQR). *Dots* represent outlier observations. *Notches* signify an estimated 95 % confidence interval around the median. *P*-values estimated by Kolmogorov–Smirnov test using all GR peaks as reference distribution. **b** Frequency of GRE sequence motifs among different modes of remodeling. Modes differ significantly in their pre-existing enhancer strength, as measured by degree of H3K27Ac (**c**) and proportion of DHS (**d**). *p*-values for (**b**, **d**) calculated by Fisher’s exact test

For each GR peak, we examined a 3-kb window centered on its summit. By measuring the area under the curve, we were able to capture changes in ChIP-Seq signal reflecting both the height and width of the signal peak. We first evaluated the GR binding and enhancer properties of sites belonging to each mode of nucleosome remodeling. The degree of GR binding differs dramatically between modes (Fig. 5a). Sites of phased remodeling have very large GR peaks and more commonly contain a canonical glucocorticoid response element (GRE, Fig. 5b). On average, sites of minimal remodeling have small GR peaks, yet the range of peak sizes is very broad.

Prior to GR binding, the enhancers belonging to each mode of remodeling also have very different properties. In unstimulated cells, regions that will later demonstrate non-central remodeling have the highest H3K27Ac and most frequent DHS. In contrast, sites of minimal remodeling have very low H3K27Ac and infrequent DHS (Fig. 5c, d). Given that H3K27Ac and DHS are widely considered to be markers of active enhancers, it is interesting to note that the size of the GR binding peak correlates poorly with the “activation state” of the enhancer to which it binds (*r*^2^ = 0.18, data not shown).

Heatmaps in Fig. 4a–d illustrate the histone modification signal in unstimulated cells (NoStim H3K4methyls) corresponding to the *k*-means clusters described above. Using histone modification signal to approximate nucleosome position, as in Kundaje et al. [7], we see that sites in every cluster have highly variable position of the signal maximum relative to the future GR binding site, that many sites demonstrate well-positioned and phased nucleosomes prior to GR binding, and that signal is again asymmetric. At a minority of sites, a peak of H3K4methyls signal occupies the site of future GR binding, suggesting a well-positioned nucleosome that is displaced by GR. Strikingly, evidence of phased nucleosome positioning can be seen in the majority of clusters (Fig. 4b, red box), but pre-existing phased positioning is not more prevalent among sites showing phased remodeling. In other words, the pattern of nucleosome remodeling correlates poorly with the pattern of nucleosome position at enhancers prior to GR binding.

### Central nucleosome remodeling increases positioning of flanking nucleosomes and suggests nucleosome eviction

Nucleosome remodeling that overlaps transcription factor binding, which we call “central” remodeling, has been previously described. For example, in their study of AR-modulated nucleosome remodeling, He et al. describe nucleosome remodeling using changes in H3K4me2 signal. In their analysis, three well-positioned nucleosomes having similar occupancy were present prior to androgen stimulation, one directly overlying, and two immediately flanking the AR binding site. AR binding was shown to result in destabilization of the central nucleosome and stabilization of flanking nucleosomes without changes in flanking nucleosome position [12, 18].

**Fig. 6:**
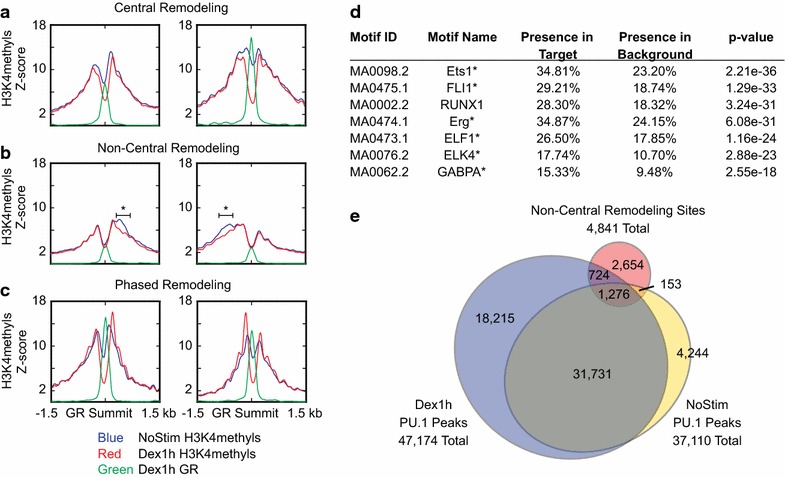
Nucleosome positioning and occupancy changes in modes of remodeling. Overlaid signal profiles of unstimulated (*blue*) and dexamethasone-stimulated (*red*) H3K4methyls as well as GR signal (*green*) in two representative clusters having central remodeling (**a**), non-central remodeling (**b**), or phased remodeling (**c**). *Asterisk symbol* in **b** denotes the regions used for motif analysis of DNA sequences associated with non-central nucleosome eviction (**d**). *Asterisk symbol* in **d** denotes members of the ETS family of transcription factors. **e** Venn diagram of regions of non-central remodeling relative to PU. 1 ChIP-Seq peaks in unstimulated and dexamethasone-stimulated cells

Our clustering analysis reveals that considerable diversity of nucleosome position, occupancy, and remodeling underlies the central mode of remodeling. GR binding diminishes occupancy of one to three underlying nucleosomes. Flanking nucleosomes become more precisely positioned and have stable or decreased occupancy (Fig. 6a, left panel). In many cases, flanking nucleosomes also undergo positional shifts, widening the NDR to accommodate GR binding (Fig. 6a, right panel). In aggregate, we see diminished central ChIP-Seq signal without corresponding increases in signal of flanking nucleosomes, suggesting that nucleosome eviction, rather than repositioning, predominates within this central mode of remodeling.

### Non-central remodeling and cooperating transcription factors

Our clustering analysis reveals a large number of GR binding sites having changes in the deltaH3K4methyls signal that do not overlap the GR peak, but occur within 1500 bp. As seen in Fig. 3, this “non-central” remodeling occurs asymmetrically, at variable distances from the GR binding site, in nearly half of GR binding instances (49 %). Sites of non-central remodeling increase the positioning of flanking nucleosomes without increasing their occupancy, again suggesting nucleosome eviction (Fig. 6b). We hypothesized that the asymmetry of non-central remodeling might have functional significance. In other words, we hypothesized that non-central remodeling accompanies the binding of one or more GR cofactors and that choice of cofactor is impacted by DNA sequence. For each individual GR binding region demonstrating non-central remodeling (denoted by asterisk symbol in Fig. 6b), we extracted the DNA sequence(s) having decreased H3K4methyls signal without colocalized GR binding. Motif analysis of these sequences shows they are highly significantly enriched for ETS family and RUNX1 transcription factor binding motifs (Fig. 6d). Several members of the ETS family have previously been reported to bind GR and other nuclear hormone receptors and are thought to serve both pioneering and tethering functions [27, 28].

We sought to validate that sites of non-central nucleosome remodeling are inducibly bound by a GR cofactor matching the predicted motifs. We performed ChIP-Seq for PU.1 (SPI1), which is essential for B cell differentiation, and one of the most highly expressed members of the ETS family in pre-B cells [29]. The overlap between non-centrally remodeled regions and PU.1 binding sites is illustrated in Fig. 6e. It is difficult to determine the biological or statistical significance of this degree of dexamethasone-induced PU.1 binding at these sites. To attempt this, we looked at all ETS binding motifs lying within the 3-kb window surrounding all GR summits. We then subtracted the regions binding GR itself. PU.1 exhibits dexamethasone-inducible binding at 1.9 % of all ETS motifs, but 4.5 % of the subset of ETS motifs having non-central nucleosome remodeling. These findings are consistent with our hypothesis that decreases in H3K4methyls signal largely reflect transcription factor binding, both for GR and PU.1, and perhaps more generally. Several additional members of the ETS family of transcription factors are expressed in B cells, and these may account for an even larger proportion of the non-centrally remodeled regions.

### Phased nucleosome remodeling and nucleosome repositioning

Approximately 6 % of GR binding sites demonstrate a pattern of remodeling that can also be described as “phased”, where the deltaK4methyls signal shows distinct, alternating peaks and valleys. Phased remodeling sites are not the most highly “activated” enhancers (Fig. 5b, c), having lower H3K27Ac and DHS than sites of non-central remodeling. Nonetheless, they have the largest GR peaks, as well as increased width and depth of the central nucleosome-depleted region (Figs. 5a, 6c). The phased appearance of remodeling reflects shifts in the position of two or more nucleosomes flanking the GR binding site. These flanking nucleosomes also become more precisely positioned and show stable or increased H3K4methyls signal, implying that phased remodeling reflects nucleosome repositioning, rather than eviction. Unlike other modes of remodeling, the total H3K4methyls signal at sites of phased remodeling increases, suggesting recruitment of histone methyltransferases or displacement of histone demethylases (data not shown).

### Minimal remodeling and de novo enhancer development

Regions lacking enhancer marks comprise the majority of sites having minimal remodeling upon GR binding (57 %). The remaining regions of minimal remodeling also have low levels of enhancer activation, bearing less H3K27Ac and DHS relative to other modes of remodeling (Fig. 5b, c). Given their relatively small GR binding peaks and low pre-existing enhancer activity, we postulated that sites of minimal remodeling could include sites of pioneering by GR, areas of GR ChIP-Seq artifact, or a mixture of both. In light of this concern, it is important to recognize that the weak histone modification signals seen prior to GR binding severely limit the sensitivity of our deltaH3K4methyls approach to detecting nucleosome remodeling. Notably, a substantial minority of minimally remodeled GR binding sites (32 %) specifically gain H3K4 methylation at nucleosomes flanking the GR peak, suggesting that these regions may be developing into de novo enhancers. Such developing enhancers could be considered “GR-specific”, defined only in response to GR binding.

### Phased nucleosome remodeling is associated with rapid enhancer activation

**Fig. 7:**
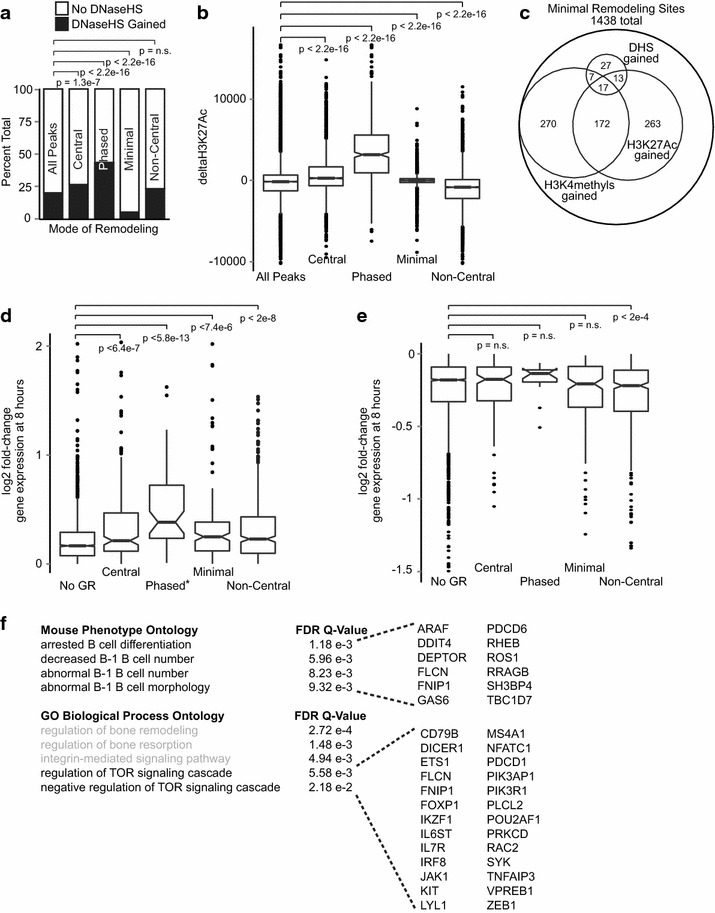
Modes of nucleosome remodeling have functionally distinct effects on enhancer activation and gene expression. Enhancer activation differs significantly between remodeling modes, as measured by proportion of sites gaining DHS (**a**) and degree of change in H3K27Ac (**b**). **c** Venn diagram of minimally remodeled GR binding sites and subsets which gain H3K4methylation, H3K27Ac, or DHS suggesting de novo enhancer development. **d** *Box and whisker plot* of log_2_ fold-change expression for genes induced following dexamethasone treatment. *Asterisk symbol* indicates that phased remodeling is associated with significantly greater induction of gene expression than other modes of remodeling. **e** *Box and whisker plot* of log_2_ fold-change expression for genes repressed following dexamethasone treatment. For **d** and **e**, *p*-values estimated by Mann–Whitney test. *n.s.* denotes *p*-value not significant. **f** GREAT (genomic regions enrichment of annotations tool) [30] analysis of GR sites having minimal remodeling. *Gray text* denotes gene ontologies not relevant to this cell type. Genes belonging to overlapping enrichment terms are listed on the *right*

We next asked whether the modes of nucleosome remodeling are associated with differential enhancer activation by measuring changes in DHS and H3K27Ac at GR binding sites (Fig. 7a, b). Both measures of enhancer activation demonstrate significant differences between modes of remodeling. Phased remodeling occurs at the most “dynamic” enhancers, gaining the largest proportion of DHS and showing the greatest gains in H3K27Ac. Central remodeling is associated with modest gains in DHS. Although some enhancers having central remodeling gain degrees of H3K27Ac similar to the most highly activated phased remodeling sites, on average, central remodeling is associated with modest increases in H3K27Ac. Despite occurring at the most highly activated pre-existing enhancers, non-central remodeling regions gain relatively little DHS, and the majority of sites show decreased H3K27Ac. Sites of minimal remodeling show very little change in DHS and relatively small gains and losses of H3K27Ac. Upon closer examination, we find that 32 % of minimal remodeling sites specifically gain H3K27Ac at nucleosomes flanking the GR peak. Altogether, more than half of all minimal remodeling regions gain one or more enhancer-associated marks (53 %, Fig. 7c), further stimulating our interest in the import of these regions as emerging enhancers.

### Modes of nucleosome remodeling have functionally distinct effects on gene expression

Finally, we wished to evaluate the relationships between GR-associated nucleosome remodeling and changes in gene expression. We measured transcript abundance by microarray at 0, 8, and 24 h after stimulation with dexamethasone. We used the assumption that each GR binding peak is most likely to modulate expression of the nearest gene (transcriptional start site within 500 kb). This distance constraint associates nearly all peaks with a gene (95 %), and many genes have multiple associated peaks (40 %). For genes associated with multiple proximal GR peaks, we considered only those genes for which all peaks have the same mode of remodeling. As seen in Fig. 7d, e, many genes that do not show proximal GR binding at the 1-hour ChIP-Seq time point are differentially expressed at the 8-hour microarray time point. This is consistent with known roles of many additional transcription factor pathways in the glucocorticoid response as well as probable changes in GR binding at later time points.

Phased remodeling is associated with the most significant induction of gene expression, and very few genes (<9 %) having proximal phased sites show decreased gene expression. Central, non-central, and minimal remodeling are also significantly associated with induction of gene expression relative to genes without proximal GR peaks. Remarkably, genes associated with minimally remodeled sites are induced as strongly as those having central or non-central remodeling. In contrast, only non-central remodeling is associated with genes repressed more significantly than genes having no proximal GR binding. In examining gene expression at the 24-h time point, we again find that each mode of remodeling remains significantly associated with induced gene expression, but none are associated with repression.

Finally, we used the Genomic Regions Enrichment of Annotations Tool (GREAT, [30]) to apply gene expression-based gene ontologies in a “pathway” analysis of GR binding sites for each mode of remodeling. The peaks associated with phased, central, or non-central remodeling each yield a large number of similar enrichment terms most notable for lymphocyte activation (Additional file 1). However, GREAT analysis of minimally remodeled peaks yields a small and distinctive subset of ontology terms that include decreased B cell number, B cell differentiation, and inhibition of the mTOR signaling pathway (Fig. 7f). This remarkable pathway specificity supports our hypothesis that the minimally remodeled GR binding sites, containing de novo and “GR-specific” enhancers, modulate expression of a distinctive subset of genes. Moreover, the associated genes and pathways have been previously shown to modulate both glucocorticoid-induced cell death and the effectiveness of ALL therapy [31, 32].

## Conclusions

Many groups have demonstrated well-positioned nucleosomes in relation to transcriptional start sites and RNA polymerase II binding in eukaryotic cells [4–7, 17, 33]. More recently, well-positioned nucleosomes have also been described at mammalian enhancers and in relation to transcription factor binding sites [6, 7]. These elegant and far-reaching studies have established a paradigm of precise nucleosomal organization at transcriptional regulatory elements influenced by several factors, including DNA sequence preferences for nucleosome occupancy, transcription factor binding, and “statistical packing”.

Much less is understood about the dynamics of nucleosome organization and their relationships to transcriptional regulation. He et al. investigated nucleosome dynamics relative to chromatin binding by androgen receptor and used H3K4me2 ChIP-Seq to describe nucleosome depletion overlapping transcription factor binding that is similar to the “central” mode of remodeling we define here [12]. This change in ChIP-Seq signal approximates increased DHS, which correlates with enhancer activation and induction of gene expression. Here we describe an approach to analyzing ChIP-Seq data that reveals considerably more complexity to nucleosome dynamics at transcription factor binding sites than has been previously appreciated. We have identified four functionally distinct modes of nucleosome remodeling that we have dubbed “central”, “non-central”, “phased”, and “minimal”.

We find that sites of central remodeling are characterized by (1) predominant eviction, rather than repositioning, of nucleosomes; (2) increased positioning of flanking nucleosomes, and (3) frequent shifts in position of flanking nucleosomes. Non-central remodeling sites are defined by GR-proximal nucleosome eviction not overlapping the GR peak. We propose that this nucleosome eviction reflects binding of transcriptional cofactors, exemplified by the ETS-family member PU.1, which can serve as both pioneer factor and tethering factor for GR. Interestingly, sites of non-central remodeling are also enriched for RUNX1 binding motifs. RUNX1 translocations producing chimeric fusion proteins with the ETS-family transcription factor ETV6 are found in 20–25 % of childhood ALL and are associated with a favorable prognosis, whereas RUNX1 amplifications are seen in ~2 % of childhood ALL and are associated with high-risk disease [34, 35]. Moreover, RUNX1 and PU.1 physically interact, and cooperate to modulate corepressor and coactivator recruitment, making the potential role(s) of RUNX1 in the GR transcriptional program an intriguing avenue for future study [36].

Many sites of non-central remodeling show decreased H3K27Ac, and a subset shows modest correlation with repression of gene expression. Given the many prior studies showing association of increased H3K27Ac, enhancer activation, and induction of gene expression [37], it is tempting to speculate that loss of H3K27Ac is associated with enhancer “deactivation” and repression of gene expression. Like other groups, we find that the correlation between decreased H3K27Ac and gene expression (*r*^2^ = 0.14) is poor relative to its converse (*r*^2^ = 0.41) [38]. We think it likely that a multivariate approach will be needed to better understand enhancer characteristics closely associated with transcriptional repression.

A minority of GR binding events is accompanied by a phased pattern of nucleosome remodeling characterized by predominant repositioning, rather than eviction, of central nucleosomes. This repositioning results in increased occupancy and positioning of one or more nucleosomes flanking the GR binding site. Phased remodeling is not only associated with enhancer activation via increased H3K27Ac, but also with rapid induction of proximal gene expression. Given the previously reported dependence of dexamethasone-induced gene expression on the SWI/SNF chromatin remodeling complex [19, 20], it is tempting to speculate that SWI/SNF activity is particularly important for phased remodeling. We hope to address this hypothesis in future experiments.

Finally, we define a subset of GR binding sites characterized by minimal nucleosome remodeling that is highly enriched for non-enhancer and weak enhancer regions. Many of these regions acquire H3K4methylation, H3K27Ac, and/or DHS only upon dexamethasone stimulation and GR binding, suggesting that GR can act as a true pioneer factor. We speculate that sites of pioneering represent de novo, “GR-specific” enhancers and speculate that they are among the most important for the leukemia cell death effect of glucocorticoid treatment. Contrary to the prevailing logic, our hypothesis suggests that the critical components of glucocorticoid-mediated transcriptional modulation are among the weakest GR binding sites, weakest enhancers, and least dynamic regions of early nucleosome remodeling. However, it is consistent with observations that prolonged glucocorticoid exposure is required to induce apoptosis in many ALL cell lines [39] and that gene expression changes in glucocorticoid-treated cells show complex kinetics [40]. It will be fascinating to follow a time-course of ChIP-Seq and gene expression to understand how the evolving enhancer landscape relates to changes in transcription.

In summary, we have developed a novel method for using histone modification-directed ChIP-Seq to reveal dynamic nucleosome occupancy and position and describe, for the first time, functionally distinct modes of nucleosome remodeling upon chromatin binding by GR. Our approach can be readily adapted to study nucleosome and enhancer dynamics mediated by other transcription factors, and we look forward to a burgeoning understanding of how nucleosome remodeling affects gene expression.

## Methods

### Cell lines and cell culture

The RS4;11 cell line was obtained from ATCC (CRL-1873). Cultures were maintained at a density of 1.5–3 × 10^6^ cells/mL of medium (RPMI, 10 % FBS, 1 % penicillin/streptomycin). Two days prior to each experiment, cells were transferred to RPMI medium containing 10 % charcoal-dextran treated serum (CDT, Omega), again supplemented with 1 % penicillin/streptomycin. For dexamethasone treatment, cells were adjusted to a density of 2 × 10^6^ cells/mL in CDT-containing medium and treated by addition of 1 mM dexamethasone (Sigma) dissolved in 100 % ethanol for a final dexamethasone concentration of 10 nM and a final ethanol content of 0.001 %. Dexamethasone treatment duration was 1 h for ChIP-Seq and DNase-Seq experiments.

### Gene expression analysis

For gene expression experiments, cells were treated with dexamethasone as above for 0, 8, or 24 h. Five replicates were performed for each time point. RNA was isolated using Qiagen RNeasy Plus Mini columns. Transcripts were quantitated via hybridization to Agilent hgu133a2 microarrays. Quality control assessment of microarray data was performed using the arrayQualityMetrics [41] package. Based upon these results, one of the unstimulated (0 h time point) replicates was excluded from subsequent analyses. Normalization and background correction were performed using the rma algorithm of the affy package [42]. Non-specific filtering was performed using the genefilter package [43] with a variance cutoff of 0.5, retaining 9953 of the initial 22,277 probe values. A single expression value for each gene at each time point was assigned using the mean of all corresponding probe values.

### Antibodies

For ChIP-Seq experiments, antibodies against the following targets were as follows: GR (1:1:1 mixture of Novus, NB300-731; Cell Signaling, D8H2; Abcam ab3579), PU.1 (1:1:1 mixture of Pierce, E.388.3; Cell Signaling 2266; Santa Cruz Biotechnology SC-352X), H3K4me1 (Abcam, ab8895), H3K4me2 (Millipore, 07-030), H3K4me3 (Abcam, ab8580), H3K27Ac (Abcam, ab4729).

### Chromatin immunoprecipitation, histone modifications

Isolation of nuclei was performed by sucrose gradient centrifugation as previously described [44]. Nuclei (5 × 10^7^ cells) were resuspended in 5 mL of MNase digestion buffer (50 mM Tris–HCl, 320 mM sucrose, 4 mM MgCl_2_, 1 mM CaCl_2_, pH 7.6). For digestion, 10 μL of MNase (New England Biolabs) was added and nuclei incubated at 37 °C for 2′45′′. Digestion was then stopped by addition of 100 μL of 0.5 M EDTA. Nuclei were pelleted and DNA-containing supernatant was removed. The remaining nuclear pellet was resuspended in 500 μL of 1 mM Tris–HCl, 0.2 mM EDTA, pH 7.6 and incubated at 4 °C for 30 min to extract larger DNA fragments. Nuclei were again pelleted and this supernatant was combined with the previous one. 100 μL of supernatant was removed from each sample and saved as input DNA for later analysis. The remaining supernatant was diluted eightfold using N-ChIP dilution buffer (50 mM Tris–HCl, 50 mM NaCl, 5 mM EDTA, pH 7.5; protease inhibitors). One-fourth of the diluted supernatant was used for each IP by addition of 20 μL antibody-coated protein G Dynabeads (Invitrogen) followed by incubation overnight at 4 °C. Chromatin-containing beads were isolated by magnetic separation and serially washed with 1 mL of cold (1) low-salt wash buffer (20 mM Tris–HCl, 150 mM NaCl, 2 mM EDTA, 0.1 % SDS, 1 % Triton X-100, pH 8.1; protease inhibitors), (2) high-salt wash buffer (20 mM Tris–HCl, 500 mM NaCl, 2 mM EDTA, 0.1 % SDS, 1 % Triton X-100, pH 8.1; protease inhibitors), (3) LiCl wash buffer (10 mM Tris–HCl, 250 mM LiCl, 1 mM EDTA, 1 % IGEPAL-CA630, 1 % sodium deoxycholate, pH 8.1; protease inhibitors), and (4) Tris–EDTA buffer. Beads were resuspended in 100 μL of elution buffer (100 mM NaHCO_3_, 1 % SDS). Input and ChIP samples were then subjected to RNase digestion by addition of 4 μL of 5 M NaCl and 1 μL DNase-free RNase A (10 mg/mL; Life Technologies) with incubation at 56 °C for 30 min. Protein digestion was then accomplished by addition of 1 μL proteinase K (NEB) and further incubation at 56 °C for 1 h. DNA was isolated using Qiagen MinElute PCR Purification Kit per manufacturer’s directions.

### Chromatin immunoprecipitation, transcription factors

For ChIP of transcription factor targets (GR, PU. 1), cells were resuspended in fixing buffer (50 mM HEPES, 100 mM NaCl, 0.8 mM EDTA, 0.5 mM EGTA, pH 7.6; protease inhibitors) at a concentration of 5 × 10^6^ cells/mL. Fixation was accomplished by addition of EGS (ethylene glycol-bis(succinic acid *N*-hydroxysuccinimide ester), 1.5 mM final concentration, Sigma) with incubation at room temperature for 30 min, followed by addition of formaldehyde (1 % final v/v ratio), and incubation at room temperature for 10 min. Quenching and isolation of nuclei were performed as previously described [45]. Nuclei were resuspended in cold sonication buffer (50 mM Tris–HCl, 1 mM EDTA, 0.5 % SDS, pH 7.6) at a concentration of 75 × 10^6^ cells/mL. 25 × 10^6^ cells were placed in each sonication tube (Covaris, Cat. 520056) and sonicated using a Covaris E220 Focused Ultrasonicator (duty cycle 20 %, intensity 10, pulse time 3 min × 6 pulses). 10 μL of sonicated chromatin was removed from each sample and saved as input DNA for later analysis. Sonicated samples were then diluted fivefold with RIPA buffer. Immunoprecipitation was done by addition of 100 μL antibody-coated protein G Dynabeads (Invitrogen) followed by incubation overnight at 4 °C. Chromatin-containing beads were isolated by magnetic separation and serially washed three times with 1 mL of cold high-salt LiCl buffer (100 mM Tris–HCl, 500 mM LiCl, 1 % IGEPAL-CA630, 1 % deoxycholate, pH 7.6) followed by 1 mL of cold TE. Elution, RNA digestion, and protein digestion were performed as described above. Reversal of cross-linking was done by incubation at 65 °C overnight, followed by DNA isolation as described above.

### DNase hypersensitivity

Isolation of nuclei for DNase hypersensitivity assays was performed by sucrose gradient centrifugation as described above with the addition of 0.15 mM spermine and 0.05 % spermidine to buffers. The nuclear pellet was resuspended in DNase digestion buffer (40 mM Tris–HCl, 10 mM NaCl, 6 mM MgCl_2_, 1 mM CaCl_2_, pH 7.9) at a concentration of 5 × 10^6^ cells/mL. DNase (Roche) digestion was performed using five different enzyme concentrations (40, 50, 60, 75, 90 U/mL) for each sample by incubation at 37 °C for 5 min. An additional cell sample was incubated without addition of exogenous DNase. Digestion was stopped by addition of an equal volume of stop-lysis buffer (50 mM Tris–HCl, 600 mM NaCl, 100 mM EDTA, 1 % SDS, pH 7.9). For sequencing purposes, 5 μg of genomic DNA isolated from the control sample was digested in a volume of 50 μL using 4 U/mL of DNase. This control digestion was stopped by adding 5 μL of 25 mM EDTA. DNA was isolated by phenol–chloroform extraction and ethanol precipitation.

### Library amplification and sequencing

Prior to library amplification, adequacy of DNA fragmentation (via MNase digestion, DNase digestion, or sonication) was verified by visualization on a 2 % agarose E-Gel (Life Technologies). DNA was size-selected using a Pippin Prep agarose gel system (2 % agarose, Sage Science) with an average fragment size of 150 bp for MNase-digested samples, 200 bp for sonicated samples, and 75 bp for DNase-digested samples. DNA yield was quantified using Quant-iT PicoGreen reagent (Life Technologies) and NanoDrop 3300 fluorospectrometer (Thermo Scientific). 1–2 ng of size-selected DNA was used for each library. ChIP DNA was end-repaired and adenylated as previously described [46]. Adenylated DNA fragments were ligated to Illumina TruSeq multiplexed adapters. Adapter-ligated DNA was then amplified using Phusion high-fidelity DNA polymerase (New England Biolabs) with TruSeq PCR primers (final concentration 0.5 mM) using the following thermocycler conditions: 98 °C × 30′; 7–9 cycles of 98 °C × 10′, 65 °C × 30′, 72 °C × 30′; 72 °C × 5 min. Single-end, 50 bp sequencing was performed at The Center for Cancer Computational Biology (Dana-Farber Cancer Institute, Boston, MA) using an Illumina HiSeq instrument.

### Data analysis

Sequences were aligned to the hg19 genome assembly with Bowtie 0.12.8 allowing one sequence mismatch and requiring unique alignments [47]. Peak-calling was performed with MACS1.4 using default settings, including unique read requirement and *p* value threshold of 1 × 10^−5^ [48]. Genome distribution of GR ChIP-Seq peaks was analyzed using the CEAS package [49]. Non-normalized ChIP-Seq signal files (.wig format) were also generated using MACS1.4. Signal files were then normalized using the ZScore tool of the Java Genomics Toolkit (JGK), creating wig files having global mean of 0 and global standard deviation of 1 [50]. These normalized ChIP-Seq signal files were used for all subsequent analyses. deltaH3K4me1, deltaH3K4me2, deltaH3K4me3, and deltaH3K27Ac files were created using the JGK Subtract tool and the deltaK4methyls file was created using the JGK Add tool. For visualization purposes, signal files were smoothed using the JGK GaussianSmooth tool with standard deviation of 20 bp. Motif analyses were performed using Haystack [51]. Area-under-the-curve measurements were performed using the WiggleTools package [52]. Gene ontology analyses were performed using the GREAT package [30].

*K*-means clustering was performed using the computeMatrix and heatmapper functions of deepTools [53]. We first estimated an appropriate *k* value by plotting the total within-groups sum of squares against the number of centroids, using a range of 5–80 centroids. Based upon this curve, we estimated that 25–30 centroids would provide a good fit to the data. As described by Kundaje et al. [7], we found that “anchoring” our signal window around the summit of the GR ChIP-Seq peak, rather than the center of a canonical GR binding motif, generated more reproducible clustering results.

In generating figures, we used the multiIntersectBed tool of the BEDTools2 package [54] and eulerAPE [55] to create Venn diagrams. Genome browser snapshots were generated using the Integrated Genome Viewer (IGV) [56]. Heatmaps and signal profiles were generated using deepTools. All other charts were generated using the ggplot2 package [57]. Statistical tests were performed using the stats package [58].

## Availability of supporting data

All microarray and sequencing data have been deposited in GEO (and SRA) with reference series GSE71617, comprised of subseries GSE71615 (microarray) and GSE71616 (ChIP-Seq and DHS).

## Additional file

10.1186/s13072-015-0046-0 GREAT output tables for the Gene Ontology (GO) and Mouse Phenotype ontologies (Excel format). GREAT analysis using all GR ChIP-Seq peaks (a), as well as subsets of GR peaks with central remodeling (b), minimal remodeling (c), non-central remodeling (d), or phased remodeling (e).

## Abbreviations

Ac: Acetyl
ALL: Acute lymphoblastic leukemia
AR: Androgen receptor
ChIP: Chromatin immunoprecipitation
DHS: DNase I hypersensitivity
ER: Estrogen receptor
GR: Glucocorticoid receptor
GREAT: Genomic regions enrichment of annotations tool
H3K4: Histone 3 lysine 4
H3K27: Histone 4 lysine 27
Me: Methyl
MNase: Micrococcal nuclease
NDR: Nucleosome-depleted region
